# 1′-Acetyl-3-phenyl-6-oxa-4-thia-2-aza­spiro­[bicyclo­[3.2.0]hept-2-ene-7,3′-indolin]-2′-one

**DOI:** 10.1107/S1600536810031016

**Published:** 2010-08-11

**Authors:** Hoong-Kun Fun, Jia Hao Goh, Yang Liu, Yan Zhang

**Affiliations:** a X-ray Crystallography Unit, School of Physics, Universiti Sains Malaysia, 11800 USM, Penang, Malaysia; b School of Chemistry and Chemical Engineering, Nanjing University, Nanjing 210093, People’s Republic of China

## Abstract

In the title indoline compound, C_19_H_14_N_2_O_3_S, the pyrrolidine ring adopts an envelope conformation with the four-connected (spiro) C atom as the flap [displacement = 0.148 (3) Å]. The mean plane formed through the indoline unit is inclined at dihedral angles of 89.92 (16) and 59.54 (12)° with the thia­zole and phenyl rings, respectively; the dihedral angle between the latter rings is 9.55 (14)°. In the crystal, pairs of inter­molecular C—H⋯O hydrogen bonds link neighbouring mol­ecules into inversion dimers, producing *R*_2_^2^(6) hydrogen-bond ring motifs. Weak inter­molecular C—H⋯π as well as π–π inter­actions [centroid–centroid distance = 3.4041 (15) Å] further consolidate the crystal structure.

## Related literature

For general background to and applications of compounds related to the title indoline compound, see: Aanandhi *et al.* (2008 ▶); Crews *et al.* (1988 ▶); Cutignano *et al.* (2001 ▶); DeRoy & Charette (2003 ▶); Gao *et al.* (2010 ▶); Kaleta *et al.* (2006 ▶); Lawrence *et al.* (2008 ▶); Muthukumar *et al.* (2008 ▶); Shi *et al.* (2010 ▶); Tsuruni *et al.* (1995 ▶); Wang *et al.* (2005 ▶); Williams *et al.* (2001 ▶); Xue *et al.* (2000 ▶); Yoshimura *et al.* (1995 ▶); Zhang *et al.* (2004 ▶). For ring conformations, see: Cremer & Pople (1975 ▶). For graph-set theory of hydrogen-bond ring motifs, see: Bernstein *et al.* (1995 ▶). For closely related structures, see: Fun *et al.* (2010 ▶); Usman *et al.* (2001 ▶, 2002 ▶). For the stability of the temperature controller used in the data collection, see: Cosier & Glazer (1986 ▶).
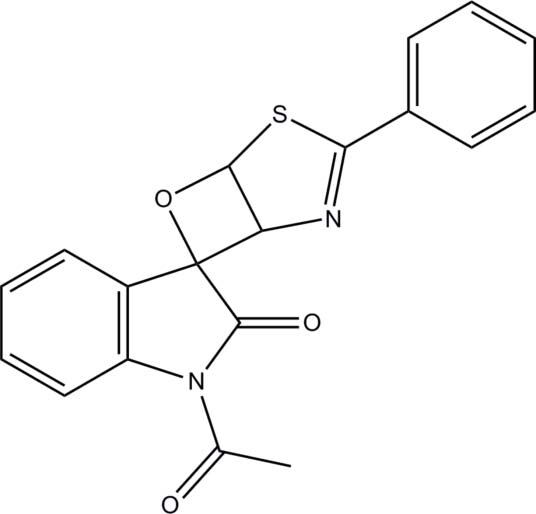


## Experimental

### 

#### Crystal data

C_19_H_14_N_2_O_3_S
*M* *_r_* = 350.38
Triclinic, 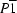

*a* = 7.5054 (3) Å
*b* = 9.4936 (3) Å
*c* = 11.6359 (4) Å
α = 103.502 (3)°
β = 91.163 (3)°
γ = 100.200 (3)°
*V* = 791.79 (5) Å^3^
*Z* = 2
Mo *K*α radiation
μ = 0.23 mm^−1^
*T* = 100 K
0.24 × 0.10 × 0.05 mm

#### Data collection

Bruker SMART APEXII CCD diffractometer
Absorption correction: multi-scan (*SADABS*; Bruker, 2009 ▶) *T*_min_ = 0.948, *T*_max_ = 0.989
10748 measured reflections
3627 independent reflections
2548 reflections with *I* > 2σ(*I*)
*R*_int_ = 0.062

#### Refinement

*R*[*F* ^2^ > 2σ(*F* ^2^)] = 0.058
*wR*(*F* ^2^) = 0.128
*S* = 1.05
3627 reflections
227 parameters
H-atom parameters constrained
Δρ_max_ = 0.35 e Å^−3^
Δρ_min_ = −0.42 e Å^−3^

### 

Data collection: *APEX2* (Bruker, 2009 ▶); cell refinement: *SAINT* (Bruker, 2009 ▶); data reduction: *SAINT*; program(s) used to solve structure: *SHELXTL* (Sheldrick, 2008 ▶); program(s) used to refine structure: *SHELXTL*; molecular graphics: *SHELXTL*; software used to prepare material for publication: *SHELXTL* and *PLATON* (Spek, 2009 ▶).

## Supplementary Material

Crystal structure: contains datablocks global, I. DOI: 10.1107/S1600536810031016/hb5595sup1.cif

Structure factors: contains datablocks I. DOI: 10.1107/S1600536810031016/hb5595Isup2.hkl

Additional supplementary materials:  crystallographic information; 3D view; checkCIF report

## Figures and Tables

**Table 1:** Hydrogen-bond geometry (Å, °) *Cg*1 is the centroid of C1–C6 phenyl ring.

*D*—H⋯*A*	*D*—H	H⋯*A*	*D*⋯*A*	*D*—H⋯*A*
C10—H10*A*⋯O1^i^	0.98	2.56	3.261 (3)	129
C14—H14*A*⋯*Cg*1^ii^	0.93	2.67	3.423 (3)	139

Symmetry codes: (i) 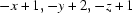; (ii) 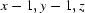.

**Fig. 1.**
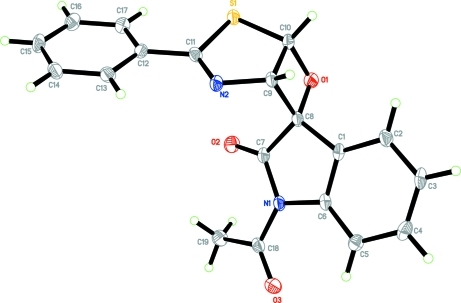
The asymmetric unit of (I) with displacement ellipsoids for non-hydrogen atoms are drawn at the 50 % probability level.

**Fig. 2.**
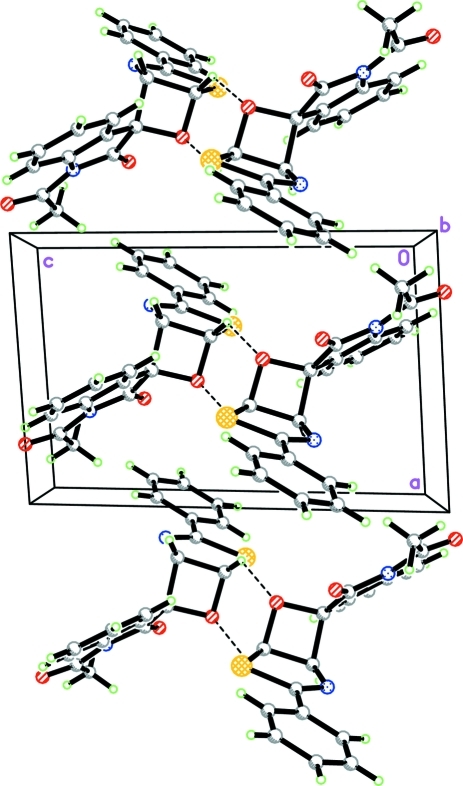
The crystal structure of (I), viewed along the b axis, showing adjacent molecules being linked into dimers. Intermolecular hydrogen bonds are shown as dashed lines.

## supplementary crystallographic information

### Comment

Oxoindole and spiroindole are important heterocyclic compounds with diverse bioactivities (Aanandhi *et al.*, 2008; Muthukumar *et al.*, 2008; Lawrence *et al.*, 2008). Photoreactions of *N*-acetylisatin with alkenes or oxazoles are convenient ways to construct spiroindole frameworks (Wang *et al.*, 2005; Zhang *et al.*, 2004; Xue *et al.*, 2000). Thiazole-containing compounds, such as the mycothiazole  (Crews *et al.*, 1988; Cutignano *et al.*,2001), cystothiazole A (Williams *et al.*,2001; DeRoy & Charette,2003) and WS75624 B (Yoshimura *et al.*,1995; Tsuruni *et al.*,1995) have attracted considerable interest due to their potential application as bio-active species. Synthesis of organic molecules containing thiazole moieties therefore has been of current research interest (Gao *et al.*,2010; Shi *et al.*,2010; Kaleta *et al.*,2006). The title compound, (I), which contains spiroindole and thiazole rings is now described.

In the title indoline compound (Fig. 1), the pyrrolidine ring (C1/C6/N1/C7/C8) of the indoline moiety adopts an envelope conformation with the C8 atom as the flap atom; the puckering parameters are Q = 0.090 (3) Å and φ = 106.4° (Cremer & Pople, 1975). The essentially planar thiazole ring (C9/C10/S1/C11/N2) and C12-C17 phenyl ring are inclined at dihedral angles of 89.92 (16) and 59.54 (12)°, respectively, with respect to the mean plane formed through the indoline moiety (C1-C8/N1). The geometric parameters agree well with those observed in the closely related structures (Fun *et al.*, 2010; Usman *et al.*, 2001, 2002).

In the crystal structure (Fig. 2), pairs of intermolecular C10—H10A···O1 hydrogen bonds (Table 1) link neighbouring molecules into dimers incorporating *R*^2^_2_(6) hydrogen bond ring motifs (Bernstein *et al.*, 1995). The crystal structure is further stabilized by weak intermolecular C14—H14A···*Cg*1 (Table 1) as well as *Cg*2···*Cg*3 [*Cg*2···*Cg*3 = 3.4041 (15); symmetry code: x, y, z] interactions where *Cg*1, *Cg*2 and *Cg*3 are the centroids of C1-C6 phenyl, thiazole and pyrrolidine rings, respectively.

### Experimental

The title compound was one of the products from the photoreaction between *N*-acetylisatin and 2-phenylthiazole. The compound was purified by flash column chromatography with ethyl acetate/petroleum ether (1:4) as eluents.  Colourless blocks of (I) were obtained from slow evaporation of an acetone and petroleum ether (1:6) solution. *M.p.* 442–444 K.

### Refinement

All hydrogen atoms were placed in their calculated positions, with C—H = 0.93–0.98 Å, and refined using a riding model, with *U*_iso_(H) = 1.2 or 1.5*U*_eq_(C). The rotating group model was applied to the methyl group.

### Crystal data

**Table:**

C_19_H_14_N_2_O_3_S	*Z* = 2
*M**_r_* = 350.38	*F*(000) = 364
Triclinic, *P*1	*D*_x_ = 1.470 Mg m^−^^3^
Hall symbol: -P 1	Mo *K*α radiation, λ = 0.71073 Å
*a* = 7.5054 (3) Å	Cell parameters from 2361 reflections
*b* = 9.4936 (3) Å	θ = 2.5–29.9°
*c* = 11.6359 (4) Å	µ = 0.23 mm^−^^1^
α = 103.502 (3)°	*T* = 100 K
β = 91.163 (3)°	Block, colourless
γ = 100.200 (3)°	0.24 × 0.10 × 0.05 mm
*V* = 791.79 (5) Å^3^	

### Data collection

**Table:**

Bruker SMART APEXII CCD diffractometer	3627 independent reflections
Radiation source: fine-focus sealed tube	2548 reflections with *I* > 2σ(*I*)
graphite	*R*_int_ = 0.062
φ and ω scans	θ_max_ = 27.5°, θ_min_ = 1.8°
Absorption correction: multi-scan (*SADABS*; Bruker, 2009)	*h* = −9→9
*T*_min_ = 0.948, *T*_max_ = 0.989	*k* = −12→10
10748 measured reflections	*l* = −15→15

### Refinement

**Table:**

Refinement on *F*^2^	Primary atom site location: structure-invariant direct methods
Least-squares matrix: full	Secondary atom site location: difference Fourier map
*R*[*F*^2^ > 2σ(*F*^2^)] = 0.058	Hydrogen site location: inferred from neighbouring sites
*wR*(*F*^2^) = 0.128	H-atom parameters constrained
*S* = 1.05	*w* = 1/[σ^2^(*F*_o_^2^) + (0.0437*P*)^2^ + 0.6382*P*] where *P* = (*F*_o_^2^ + 2*F*_c_^2^)/3
3627 reflections	(Δ/σ)_max_ < 0.001
227 parameters	Δρ_max_ = 0.35 e Å^−^^3^
0 restraints	Δρ_min_ = −0.42 e Å^−^^3^

### Special details

**Table:**

Experimental. The crystal was placed in the cold stream of an Oxford Cryosystems Cobra open-flow nitrogen cryostat (Cosier & Glazer, 1986) operating at 100.0 (1)K.
Geometry. All esds (except the esd in the dihedral angle between two l.s. planes) are estimated using the full covariance matrix. The cell esds are taken into account individually in the estimation of esds in distances, angles and torsion angles; correlations between esds in cell parameters are only used when they are defined by crystal symmetry. An approximate (isotropic) treatment of cell esds is used for estimating esds involving l.s. planes.
Refinement. Refinement of F^2^ against ALL reflections. The weighted R-factor wR and goodness of fit S are based on F^2^, conventional R-factors R are based on F, with F set to zero for negative F^2^. The threshold expression of F^2^ > 2sigma(F^2^) is used only for calculating R-factors(gt) etc. and is not relevant to the choice of reflections for refinement. R-factors based on F^2^ are statistically about twice as large as those based on F, and R- factors based on ALL data will be even larger.

### Fractional atomic coordinates and isotropic or equivalent isotropic displacement parameters (Å^2^)

**Table:**

	*x*	*y*	*z*	*U*_iso_*/*U*_eq_	
S1	0.31424 (10)	0.63788 (7)	0.48799 (6)	0.01816 (18)	
O1	0.5459 (3)	0.90182 (19)	0.58975 (16)	0.0190 (4)	
O2	0.6257 (3)	0.7176 (2)	0.74453 (17)	0.0244 (5)	
O3	0.8183 (3)	0.9955 (2)	1.07309 (17)	0.0229 (5)	
N1	0.6758 (3)	0.9398 (2)	0.89013 (19)	0.0156 (5)	
N2	0.2265 (3)	0.7250 (2)	0.70929 (19)	0.0158 (5)	
C1	0.5502 (4)	1.0821 (3)	0.7864 (2)	0.0161 (6)	
C2	0.5067 (4)	1.2098 (3)	0.7631 (2)	0.0186 (6)	
H2A	0.4462	1.2074	0.6919	0.022*	
C3	0.5566 (4)	1.3413 (3)	0.8498 (3)	0.0204 (6)	
H3A	0.5333	1.4291	0.8358	0.025*	
C4	0.6407 (4)	1.3411 (3)	0.9567 (3)	0.0212 (6)	
H4A	0.6693	1.4293	1.0143	0.025*	
C5	0.6845 (4)	1.2135 (3)	0.9816 (2)	0.0180 (6)	
H5A	0.7404	1.2149	1.0540	0.022*	
C6	0.6399 (4)	1.0849 (3)	0.8924 (2)	0.0159 (6)	
C7	0.6062 (4)	0.8429 (3)	0.7805 (2)	0.0173 (6)	
C8	0.5034 (4)	0.9279 (3)	0.7127 (2)	0.0161 (6)	
C9	0.2983 (4)	0.8603 (3)	0.6760 (2)	0.0156 (6)	
H9A	0.2192	0.9334	0.6934	0.019*	
C10	0.3596 (4)	0.8344 (3)	0.5480 (2)	0.0168 (6)	
H10A	0.3038	0.8885	0.4995	0.020*	
C11	0.2314 (4)	0.6112 (3)	0.6260 (2)	0.0156 (6)	
C12	0.1634 (4)	0.4594 (3)	0.6374 (2)	0.0157 (6)	
C13	0.0699 (4)	0.4403 (3)	0.7366 (2)	0.0180 (6)	
H13A	0.0553	0.5223	0.7949	0.022*	
C14	−0.0011 (4)	0.3003 (3)	0.7486 (2)	0.0198 (6)	
H14A	−0.0637	0.2881	0.8148	0.024*	
C15	0.0213 (4)	0.1773 (3)	0.6613 (3)	0.0213 (6)	
H15A	−0.0262	0.0829	0.6691	0.026*	
C16	0.1142 (4)	0.1963 (3)	0.5632 (3)	0.0217 (6)	
H16A	0.1287	0.1142	0.5050	0.026*	
C17	0.1866 (4)	0.3371 (3)	0.5504 (2)	0.0181 (6)	
H17A	0.2497	0.3491	0.4844	0.022*	
C18	0.7757 (4)	0.9051 (3)	0.9798 (2)	0.0170 (6)	
C19	0.8273 (4)	0.7560 (3)	0.9534 (3)	0.0240 (7)	
H19D	0.8923	0.7446	1.0216	0.036*	
H19A	0.9029	0.7467	0.8875	0.036*	
H19B	0.7197	0.6812	0.9343	0.036*	

### Atomic displacement parameters (Å^2^)

**Table:**

	*U*^11^	*U*^22^	*U*^33^	*U*^12^	*U*^13^	*U*^23^
S1	0.0253 (4)	0.0130 (3)	0.0158 (3)	0.0014 (3)	0.0011 (3)	0.0044 (3)
O1	0.0228 (11)	0.0163 (9)	0.0174 (10)	0.0026 (8)	0.0010 (8)	0.0037 (8)
O2	0.0338 (13)	0.0134 (10)	0.0266 (11)	0.0091 (9)	−0.0044 (9)	0.0030 (8)
O3	0.0289 (12)	0.0223 (10)	0.0180 (10)	0.0053 (9)	0.0006 (9)	0.0060 (9)
N1	0.0201 (13)	0.0111 (10)	0.0167 (11)	0.0030 (9)	0.0020 (9)	0.0054 (9)
N2	0.0177 (12)	0.0115 (11)	0.0184 (12)	0.0027 (9)	0.0012 (9)	0.0042 (9)
C1	0.0174 (15)	0.0109 (12)	0.0202 (14)	0.0008 (11)	0.0042 (11)	0.0052 (11)
C2	0.0188 (15)	0.0152 (13)	0.0237 (14)	0.0041 (11)	−0.0008 (12)	0.0081 (11)
C3	0.0198 (15)	0.0101 (13)	0.0332 (16)	0.0044 (11)	0.0041 (12)	0.0074 (12)
C4	0.0237 (17)	0.0133 (13)	0.0241 (15)	0.0022 (12)	0.0052 (12)	0.0002 (11)
C5	0.0167 (15)	0.0166 (13)	0.0196 (14)	0.0016 (11)	0.0023 (11)	0.0034 (11)
C6	0.0167 (15)	0.0125 (12)	0.0204 (14)	0.0027 (11)	0.0025 (11)	0.0075 (11)
C7	0.0210 (16)	0.0120 (13)	0.0192 (14)	0.0026 (11)	0.0010 (11)	0.0050 (11)
C8	0.0226 (16)	0.0111 (12)	0.0151 (13)	0.0020 (11)	0.0023 (11)	0.0049 (11)
C9	0.0196 (15)	0.0118 (12)	0.0162 (13)	0.0018 (11)	0.0015 (11)	0.0055 (11)
C10	0.0193 (15)	0.0116 (12)	0.0202 (14)	0.0018 (11)	−0.0006 (11)	0.0062 (11)
C11	0.0160 (14)	0.0149 (13)	0.0180 (13)	0.0045 (11)	0.0003 (11)	0.0066 (11)
C12	0.0145 (14)	0.0134 (12)	0.0191 (14)	0.0012 (11)	−0.0019 (11)	0.0049 (11)
C13	0.0235 (16)	0.0155 (13)	0.0149 (13)	0.0045 (12)	0.0013 (11)	0.0027 (11)
C14	0.0211 (16)	0.0212 (14)	0.0193 (14)	0.0032 (12)	0.0025 (12)	0.0100 (12)
C15	0.0213 (16)	0.0119 (13)	0.0314 (16)	0.0016 (11)	0.0001 (13)	0.0079 (12)
C16	0.0244 (17)	0.0136 (13)	0.0256 (15)	0.0046 (12)	0.0013 (13)	0.0011 (12)
C17	0.0175 (15)	0.0171 (14)	0.0197 (14)	0.0044 (11)	0.0029 (11)	0.0033 (11)
C18	0.0166 (15)	0.0184 (13)	0.0178 (14)	0.0017 (11)	0.0025 (11)	0.0086 (11)
C19	0.0275 (17)	0.0188 (14)	0.0282 (16)	0.0086 (12)	−0.0068 (13)	0.0083 (12)

### Geometric parameters (Å, °)

**Table:**

S1—C11	1.790 (3)	C7—C8	1.538 (4)
S1—C10	1.801 (3)	C8—C9	1.568 (4)
O1—C8	1.446 (3)	C9—C10	1.544 (4)
O1—C10	1.452 (3)	C9—H9A	0.9800
O2—C7	1.201 (3)	C10—H10A	0.9800
O3—C18	1.212 (3)	C11—C12	1.479 (4)
N1—C18	1.406 (3)	C12—C17	1.390 (4)
N1—C7	1.415 (3)	C12—C13	1.393 (4)
N1—C6	1.444 (3)	C13—C14	1.382 (4)
N2—C11	1.278 (3)	C13—H13A	0.9300
N2—C9	1.444 (3)	C14—C15	1.396 (4)
C1—C6	1.385 (4)	C14—H14A	0.9300
C1—C2	1.393 (3)	C15—C16	1.380 (4)
C1—C8	1.490 (4)	C15—H15A	0.9300
C2—C3	1.396 (4)	C16—C17	1.394 (4)
C2—H2A	0.9300	C16—H16A	0.9300
C3—C4	1.383 (4)	C17—H17A	0.9300
C3—H3A	0.9300	C18—C19	1.498 (4)
C4—C5	1.400 (4)	C19—H19D	0.9600
C4—H4A	0.9300	C19—H19A	0.9600
C5—C6	1.389 (4)	C19—H19B	0.9600
C5—H5A	0.9300		
			
C11—S1—C10	90.03 (12)	C8—C9—H9A	113.1
C8—O1—C10	92.80 (19)	O1—C10—C9	91.54 (19)
C18—N1—C7	126.1 (2)	O1—C10—S1	116.58 (17)
C18—N1—C6	124.7 (2)	C9—C10—S1	106.43 (17)
C7—N1—C6	109.1 (2)	O1—C10—H10A	113.4
C11—N2—C9	112.1 (2)	C9—C10—H10A	113.4
C6—C1—C2	121.3 (2)	S1—C10—H10A	113.4
C6—C1—C8	110.1 (2)	N2—C11—C12	122.6 (2)
C2—C1—C8	128.5 (2)	N2—C11—S1	118.4 (2)
C1—C2—C3	117.9 (3)	C12—C11—S1	118.90 (19)
C1—C2—H2A	121.0	C17—C12—C13	120.0 (2)
C3—C2—H2A	121.0	C17—C12—C11	121.5 (2)
C4—C3—C2	120.0 (2)	C13—C12—C11	118.5 (2)
C4—C3—H3A	120.0	C14—C13—C12	120.3 (2)
C2—C3—H3A	120.0	C14—C13—H13A	119.8
C3—C4—C5	122.7 (3)	C12—C13—H13A	119.8
C3—C4—H4A	118.7	C13—C14—C15	119.9 (3)
C5—C4—H4A	118.7	C13—C14—H14A	120.0
C6—C5—C4	116.4 (3)	C15—C14—H14A	120.0
C6—C5—H5A	121.8	C16—C15—C14	119.7 (2)
C4—C5—H5A	121.8	C16—C15—H15A	120.2
C1—C6—C5	121.6 (2)	C14—C15—H15A	120.2
C1—C6—N1	109.4 (2)	C15—C16—C17	120.8 (3)
C5—C6—N1	128.9 (2)	C15—C16—H16A	119.6
O2—C7—N1	126.9 (2)	C17—C16—H16A	119.6
O2—C7—C8	125.5 (2)	C12—C17—C16	119.3 (3)
N1—C7—C8	107.6 (2)	C12—C17—H17A	120.3
O1—C8—C1	117.7 (2)	C16—C17—H17A	120.3
O1—C8—C7	111.3 (2)	O3—C18—N1	119.7 (2)
C1—C8—C7	102.8 (2)	O3—C18—C19	123.0 (2)
O1—C8—C9	90.84 (19)	N1—C18—C19	117.2 (2)
C1—C8—C9	118.2 (2)	C18—C19—H19D	109.5
C7—C8—C9	116.3 (2)	C18—C19—H19A	109.5
N2—C9—C10	113.0 (2)	H19D—C19—H19A	109.5
N2—C9—C8	116.6 (2)	C18—C19—H19B	109.5
C10—C9—C8	84.8 (2)	H19D—C19—H19B	109.5
N2—C9—H9A	113.1	H19A—C19—H19B	109.5
C10—C9—H9A	113.1		
			
C6—C1—C2—C3	0.1 (4)	C11—N2—C9—C8	−94.8 (3)
C8—C1—C2—C3	176.1 (3)	O1—C8—C9—N2	114.4 (2)
C1—C2—C3—C4	−2.3 (4)	C1—C8—C9—N2	−123.2 (2)
C2—C3—C4—C5	2.1 (4)	C7—C8—C9—N2	0.0 (3)
C3—C4—C5—C6	0.4 (4)	O1—C8—C9—C10	1.18 (17)
C2—C1—C6—C5	2.4 (4)	C1—C8—C9—C10	123.6 (2)
C8—C1—C6—C5	−174.2 (3)	C7—C8—C9—C10	−113.2 (2)
C2—C1—C6—N1	−177.0 (3)	C8—O1—C10—C9	1.27 (18)
C8—C1—C6—N1	6.4 (3)	C8—O1—C10—S1	−107.98 (19)
C4—C5—C6—C1	−2.6 (4)	N2—C9—C10—O1	−117.9 (2)
C4—C5—C6—N1	176.7 (3)	C8—C9—C10—O1	−1.18 (17)
C18—N1—C6—C1	175.4 (3)	N2—C9—C10—S1	0.4 (3)
C7—N1—C6—C1	−0.3 (3)	C8—C9—C10—S1	117.15 (17)
C18—N1—C6—C5	−3.9 (4)	C11—S1—C10—O1	99.1 (2)
C7—N1—C6—C5	−179.7 (3)	C11—S1—C10—C9	−1.19 (19)
C18—N1—C7—O2	−2.6 (5)	C9—N2—C11—C12	−179.1 (2)
C6—N1—C7—O2	173.1 (3)	C9—N2—C11—S1	−2.2 (3)
C18—N1—C7—C8	178.8 (2)	C10—S1—C11—N2	2.1 (2)
C6—N1—C7—C8	−5.6 (3)	C10—S1—C11—C12	179.2 (2)
C10—O1—C8—C1	−124.1 (2)	N2—C11—C12—C17	−173.5 (3)
C10—O1—C8—C7	117.6 (2)	S1—C11—C12—C17	9.6 (3)
C10—O1—C8—C9	−1.26 (18)	N2—C11—C12—C13	8.2 (4)
C6—C1—C8—O1	−132.0 (2)	S1—C11—C12—C13	−168.7 (2)
C2—C1—C8—O1	51.7 (4)	C17—C12—C13—C14	−0.5 (4)
C6—C1—C8—C7	−9.3 (3)	C11—C12—C13—C14	177.8 (3)
C2—C1—C8—C7	174.4 (3)	C12—C13—C14—C15	0.2 (4)
C6—C1—C8—C9	120.4 (3)	C13—C14—C15—C16	−0.1 (4)
C2—C1—C8—C9	−55.9 (4)	C14—C15—C16—C17	0.2 (4)
O2—C7—C8—O1	−42.9 (4)	C13—C12—C17—C16	0.6 (4)
N1—C7—C8—O1	135.8 (2)	C11—C12—C17—C16	−177.7 (3)
O2—C7—C8—C1	−169.8 (3)	C15—C16—C17—C12	−0.4 (4)
N1—C7—C8—C1	8.9 (3)	C7—N1—C18—O3	−175.5 (3)
O2—C7—C8—C9	59.3 (4)	C6—N1—C18—O3	9.5 (4)
N1—C7—C8—C9	−122.0 (2)	C7—N1—C18—C19	5.5 (4)
C11—N2—C9—C10	1.1 (3)	C6—N1—C18—C19	−169.5 (2)

### Hydrogen-bond geometry (Å, °)

**Table:**

Cg1 is the centroid of C1–C6 phenyl ring.

**Table:**

*D*—H···*A*	*D*—H	H···*A*	*D*···*A*	*D*—H···*A*
C10—H10A···O1^i^	0.98	2.56	3.261 (3)	129
C14—H14A···Cg1^ii^	0.93	2.67	3.423 (3)	139

Symmetry codes: (i) −*x*+1, −*y*+2, −*z*+1; (ii) *x*−1, *y*−1, *z*.

## References

[bb1] Aanandhi, M. V., Vaidhyalingam, V. & George, S. (2008). *Asian J. Chem.* **20**, 4588–4594.

[bb2] Bernstein, J., Davis, R. E., Shimoni, L. & Chang, N.-L. (1995). *Angew. Chem. Int. Ed. Engl.* **34**, 1555–1573.

[bb3] Bruker (2009). *APEX2*, *SAINT* and *SADABS* Bruker AXS Inc., Madison, Wisconsin, USA.

[bb4] Cosier, J. & Glazer, A. M. (1986). *J. Appl. Cryst.* **19**, 105–107.

[bb5] Cremer, D. & Pople, J. A. (1975). *J. Am. Chem. Soc.* **97**, 1354–1358.

[bb6] Crews, P., Kakou, Y. & Quinoa, E. (1988). *J. Am. Chem. Soc.* **110**, 4365–4368.

[bb7] Cutignano, A., Bruno, I., Bifulco, G., Casapullo, A., Debitus, C., Gomez-Paloma, L. & Riccio, R. (2001). *Eur. J. Org. Chem.* pp. 775–778. 10.1021/np010053+ 11678667

[bb8] DeRoy, P. L. & Charette, A. B. (2003). *Org. Lett.* **5**, 4163–4165. 10.1021/ol035600s 14572275

[bb9] Fun, H.-K., Goh, J. H., Liu, Y. & Zhang, Y. (2010). *Acta Cryst.* E**66**, o737–o738. 10.1107/S1600536810007270 PMC2983819 21580583

[bb10] Gao, X., Pan, Y.-M., Lin, M., Chen, L. & Zhan, Z.-P. (2010). *Org. Biomol. Chem.* **8**, 3259–3266. 10.1039/c002093a 20502779

[bb11] Kaleta, Z., Makowshi, B. T., So’os, T. & Dembinski, R. (2006). *Org. Lett.* **8**, 1625–1628. 10.1021/ol060208a 16597126

[bb12] Lawrence, H. R., Pireddu, R., Chen, L., Luo, Y., Sung, S.-S., Szymanski, A. M., Yip, M. L. R., Guida, W. C., Sebti, S. M., Wu, J. & Lawrence, N. J. (2008). *J. Med. Chem.* **51**, 4948–4956. 10.1021/jm8002526 PMC2744494 18680359

[bb13] Muthukumar, V. A., George, S. & Vaidhyalingam, V. (2008). *Biol. Pharm. Bull.* **31**, 1461–1464. 10.1248/bpb.31.1461 18591793

[bb14] Sheldrick, G. M. (2008). *Acta Cryst.* A**64**, 112–122. 10.1107/S0108767307043930 18156677

[bb15] Shi, B., Blake, A. J., Lewis, W., Campbell, I. B., Judkins, B. D. & Moody, C. J. (2010). *J. Org. Chem.* **75**, 152–161. 10.1021/jo902256r 19954177

[bb16] Spek, A. L. (2009). *Acta Cryst.* D**65**, 148–155. 10.1107/S090744490804362X PMC2631630 19171970

[bb17] Tsuruni, Y., Ueda, H., Hayashi, K., Takase, S., Nishikawa, M., Kiyoto, S. & Okuhara, M. (1995). *J. Antibiot.* **48**, 1066–1072. 10.7164/antibiotics.48.1066 7490208

[bb18] Usman, A., Razak, I. A., Fun, H.-K., Chantrapromma, S., Zhang, Y. & Xu, J.-H. (2001). *Acta Cryst.* E**57**, o1070–o1072. 10.1107/s0108270101016304 11740110

[bb19] Usman, A., Razak, I. A., Fun, H.-K., Chantrapromma, S., Zhang, Y. & Xu, J.-H. (2002). *Acta Cryst.* E**58**, o37–o39.

[bb20] Wang, L., Zhang, Y., Hu, H.-Y., Fun, H. K. & Xu, J.-X. (2005). *J. Org. Chem.* **70**, 3850–3858. 10.1021/jo047870+ 15876070

[bb21] Williams, D. R., Patnaik, S. & Clark, M. P. (2001). *J. Org. Chem.* **66**, 8463–8469. 10.1021/jo0106905 11735526

[bb22] Xue, J., Zhang, Y., Wang, X.-L., Fun, H. K. & Xu, J.-X. (2000). *Org. Lett.* **2**, 2583–2586. 10.1021/ol000110a 10990402

[bb23] Yoshimura, S., Tsuruni, Y., Takase, S. & Okuhara, M. (1995). *J. Antibiot.* **48**, 1073–1075. 10.7164/antibiotics.48.1073 7490209

[bb24] Zhang, Y., Wang, L., Zhang, M., Fun, H.-K. & Xu, J.-X. (2004). *Org. Lett.* **6**, 4893–4895. 10.1021/ol048028t 15606093

